# Supplementary material for: Deletion of wfs1 Impairs Oligodendrocyte Precursor Cells Dorsal Distribution and Myelination Through the wfs1-hmgcs1 Axis in Zebrafish
Source: Biology (Basel). 2026 Aug 16;15(16):1402. doi: 10.3390/biology15161402 (PMC13510151; doi:10.3390/biology15161402)
Supplement: Supplementary file 1 [file biology-15-01402-s001.zip › File S1-Transcriptome sequencing data and differentially expressed genes (DEGs) in wfs1-- zebrafish at 5 dpf/2.KEGG/wfs1KOvsWT/GSEA/WT/OXIDATIVE_PHOSPHORYLATION/OXIDATIVE_PHOSPHORYLATION(DRE00190).html]

Details for gene set OXIDATIVE\_PHOSPHORYLATION(DRE00190)[GSEA]

|  || Dataset | deg.sample |
| Phenotype | sample.cls |
| Upregulated in class | WT |
| GeneSet | OXIDATIVE\_PHOSPHORYLATION(DRE00190) |
| Enrichment Score (ES) | -0.20838994 |
| Normalized Enrichment Score (NES) | -0.85863274 |
| Nominal p-value | 0.5443038 |
| FDR q-value | 0.7932376 |
| FWER p-Value | 1.0 |
Table: GSEA Results Summary

  

Fig 1: Enrichment plot: OXIDATIVE\_PHOSPHORYLATION(DRE00190)      
 Profile of the Running ES Score & Positions of GeneSet Members on the Rank Ordered List

  

| PROBE | DESCRIPTION (from dataset) | GENE SYMBOL | GENE\_TITLE | RANK IN GENE LIST | RANK METRIC SCORE | RUNNING ES | CORE ENRICHMENT || 1 | 573619 | ndufab1a |  |  | 14 | 4.959 | 0.0519 | No |
| 2 | 447914\_1 | cox17 |  |  | 57 | 4.862 | 0.1019 | No |
| 3 | 359840 | atp6v1b2 |  |  | 274 | 3.761 | 0.1347 | No |
| 4 | 447942 | cox6a2 |  |  | 746 | 2.477 | 0.1460 | No |
| 5 | 767750\_2 | atp5ib |  |  | 1131 | 1.967 | 0.1547 | No |
| 6 | 393764 | ndufb2 |  |  | 1769 | 1.481 | 0.1502 | No |
| 7 | 561117\_1 | atp6v0a2b |  |  | 1905 | 1.412 | 0.1608 | No |
| 8 | 445129\_1 | sdhc |  |  | 1953 | 1.389 | 0.1740 | No |
| 9 | 393793 | uqcrc1 |  |  | 2224 | 1.277 | 0.1790 | No |
| 10 | 751638 | cox5b2 |  |  | 2518 | 1.168 | 0.1821 | No |
| 11 | 359839 | atp6v1ba |  |  | 2778 | 1.087 | 0.1854 | No |
| 12 | 394181\_1 | cox15 |  |  | 2942 | 1.052 | 0.1913 | No |
| 13 | 436762 | sdhda |  |  | 3054 | 1.024 | 0.1986 | No |
| 14 | 767750 | atp5ib |  |  | 3673 | 0.897 | 0.1886 | No |
| 15 | 553384 | cox10 |  |  | 4639 | 0.729 | 0.1659 | No |
| 16 | 554100 | ndufs8b |  |  | 5088 | 0.669 | 0.1588 | No |
| 17 | 562149 | sdhb |  |  | 5566 | 0.630 | 0.1504 | No |
| 18 | 100073345 | lhpp |  |  | 6136 | 0.574 | 0.1386 | No |
| 19 | 556538\_1 | ndufa12 |  |  | 6421 | 0.544 | 0.1353 | No |
| 20 | 337583 | atp6v1ab |  |  | 6443 | 0.542 | 0.1404 | No |
| 21 | 100005158\_1 | cox11 |  |  | 6560 | 0.532 | 0.1423 | No |
| 22 | 798291 | atp5e |  |  | 6822 | 0.508 | 0.1395 | No |
| 23 | 393700 | ndufa8 |  |  | 6830 | 0.507 | 0.1446 | No |
| 24 | 322811 | atp6v0d1 |  |  | 6941 | 0.497 | 0.1464 | No |
| 25 | 394151 | atp5g3a |  |  | 7852 | 0.435 | 0.1223 | No |
| 26 | 100333135 | si:ch1073-325m22.2 |  |  | 8533 | 0.400 | 0.1051 | No |
| 27 | 100330915 | si:dkey-85n7.8 |  |  | 9079 | 0.361 | 0.0917 | No |
| 28 | 140533 | ND3 |  |  | 9782 | 0.317 | 0.0729 | No |
| 29 | 406298\_1 | ndufa4 |  |  | 10280 | 0.286 | 0.0603 | No |
| 30 | 569793 | ndufa9b |  |  | 10481 | 0.274 | 0.0568 | No |
| 31 | 393706 | ppa1a |  |  | 10720 | 0.260 | 0.0521 | No |
| 32 | 100000992 | si:ch211-235e9.6 |  |  | 10890 | 0.250 | 0.0494 | No |
| 33 | 563476 | cox7b |  |  | 10950 | 0.247 | 0.0501 | No |
| 34 | 100003139 | tcirg1a |  |  | 11269 | 0.229 | 0.0425 | No |
| 35 | 325402 | atp6v0cb |  |  | 11397 | 0.222 | 0.0409 | No |
| 36 | 140541 | COX3 |  |  | 11660 | 0.208 | 0.0348 | No |
| 37 | 796997 | ndufs7 |  |  | 11858 | 0.198 | 0.0307 | No |
| 38 | 445122 | ndufa3 |  |  | 11919 | 0.195 | 0.0308 | No |
| 39 | 393943 | ndufa6 |  |  | 12056 | 0.188 | 0.0285 | No |
| 40 | 321724 | atp6v0b |  |  | 12410 | 0.169 | 0.0192 | No |
| 41 | 140536 | ND6 |  |  | 13213 | 0.130 | -0.0047 | No |
| 42 | 140531 | ND1 |  |  | 13453 | 0.118 | -0.0110 | No |
| 43 | 445042 | ndufa7 |  |  | 13581 | 0.112 | -0.0139 | No |
| 44 | 140532 | ND2 |  |  | 13672 | 0.108 | -0.0156 | No |
| 45 | 393821 | ndufb7 |  |  | 13910 | 0.096 | -0.0220 | No |
| 46 | 767664\_1 | cox7a1 |  |  | 13979 | 0.094 | -0.0232 | No |
| 47 | 140535 | ND5 |  |  | 14336 | 0.077 | -0.0336 | No |
| 48 | 559659 | atp5ia |  |  | 14787 | 0.056 | -0.0472 | No |
| 49 | 406413 | ndufs8a |  |  | 14884 | 0.052 | -0.0497 | No |
| 50 | 561469\_1 | atp6v0a2a |  |  | 15040 | 0.044 | -0.0541 | No |
| 51 | 140539 | COX1 |  |  | 15120 | 0.041 | -0.0561 | No |
| 52 | 140540 | COX2 |  |  | 15750 | 0.009 | -0.0759 | No |
| 53 | 561469 | atp6v0a2a |  |  | 15771 | 0.008 | -0.0764 | No |
| 54 | 406306 | ndufb4 |  |  | 15811 | 0.006 | -0.0776 | No |
| 55 | 140512 | CYTB |  |  | 16039 | -0.003 | -0.0847 | No |
| 56 | 100332691 | si:ch73-173p19.2 |  |  | 16577 | -0.019 | -0.1014 | No |
| 57 | 554103 | cox7a2a |  |  | 16682 | -0.022 | -0.1045 | No |
| 58 | 573021 | ndufb9 |  |  | 17203 | -0.041 | -0.1204 | No |
| 59 | 415243 | ndufa1 |  |  | 17214 | -0.041 | -0.1203 | No |
| 60 | 100537318 | ndufv3 |  |  | 17248 | -0.043 | -0.1209 | No |
| 61 | novel.839 | - |  |  | 17355 | -0.048 | -0.1238 | No |
| 62 | 140519 | ATP6 |  |  | 17469 | -0.052 | -0.1268 | No |
| 63 | 335191 | atp5o |  |  | 17761 | -0.066 | -0.1352 | No |
| 64 | 406298 | ndufa4 |  |  | 18253 | -0.088 | -0.1498 | No |
| 65 | 562219 | ndufb11 |  |  | 18273 | -0.088 | -0.1495 | No |
| 66 | 324307 | atp6v0a1a |  |  | 18391 | -0.093 | -0.1522 | No |
| 67 | 393829 | ndufb8 |  |  | 18820 | -0.114 | -0.1645 | No |
| 68 | 550531 | ppa1b |  |  | 18868 | -0.117 | -0.1647 | No |
| 69 | 81541 | atp5g3b |  |  | 18895 | -0.118 | -0.1643 | No |
| 70 | 192336 | atp6v0ca |  |  | 18982 | -0.121 | -0.1657 | No |
| 71 | 406342 | tcirg1b |  |  | 18994 | -0.121 | -0.1648 | No |
| 72 | 100004566 | atp6ap1a |  |  | 19068 | -0.125 | -0.1658 | No |
| 73 | 569770 | cox8b |  |  | 19098 | -0.126 | -0.1654 | No |
| 74 | 553660 | ndufs5 |  |  | 19236 | -0.132 | -0.1683 | No |
| 75 | 541390 | ndufc2 |  |  | 19471 | -0.143 | -0.1742 | No |
| 76 | 541328 | ndufa5 |  |  | 19478 | -0.143 | -0.1728 | No |
| 77 | 406726 | ndufa4l |  |  | 19614 | -0.149 | -0.1755 | No |
| 78 | 140534 | ND4 |  |  | 20213 | -0.175 | -0.1925 | No |
| 79 | 415243\_1 | ndufa1 |  |  | 20329 | -0.180 | -0.1943 | No |
| 80 | 100332034 | ndufb3 |  |  | 20605 | -0.194 | -0.2009 | No |
| 81 | 554101 | cox5aa |  |  | 20691 | -0.198 | -0.2015 | No |
| 82 | 406359 | uqcrfs1 |  |  | 20779 | -0.203 | -0.2021 | No |
| 83 | 335709 | atp5d |  |  | 20785 | -0.203 | -0.2001 | No |
| 84 | 402883 | atp5j2 |  |  | 20808 | -0.204 | -0.1986 | No |
| 85 | 335774 | cox6a1 |  |  | 21080 | -0.220 | -0.2048 | No |
| 86 | 564379 | cyc1 |  |  | 21143 | -0.223 | -0.2045 | No |
| 87 | 447913 | ndufs6 |  |  | 21171 | -0.225 | -0.2029 | No |
| 88 | 566723 | uqcrh |  |  | 21287 | -0.230 | -0.2041 | No |
| 89 | 393478 | cox6b2 |  |  | 21307 | -0.231 | -0.2023 | No |
| 90 | 406599 | atp5j |  |  | 21464 | -0.239 | -0.2047 | No |
| 91 | 393700\_1 | ndufa8 |  |  | 21478 | -0.240 | -0.2026 | No |
| 92 | 100535363 | cox7a2b |  |  | 21548 | -0.243 | -0.2022 | No |
| 93 | 393781 | ndufb6 |  |  | 21625 | -0.247 | -0.2020 | No |
| 94 | 553384\_1 | cox10 |  |  | 21784 | -0.256 | -0.2043 | No |
| 95 | 325712 | ndufab1b |  |  | 21801 | -0.257 | -0.2020 | No |
| 96 | 393703 | ndufb10 |  |  | 21856 | -0.259 | -0.2010 | No |
| 97 | 393675 | atp5h |  |  | 22091 | -0.272 | -0.2055 | Yes |
| 98 | 322549 | uqcrc2b |  |  | 22169 | -0.276 | -0.2050 | Yes |
| 99 | 445291 | ndufv1 |  |  | 22170 | -0.277 | -0.2021 | Yes |
| 100 | 140538 | ND4L |  |  | 22200 | -0.278 | -0.2001 | Yes |
| 101 | 100003066 | ndufa4l2a |  |  | 22239 | -0.281 | -0.1983 | Yes |
| 102 | 393687 | ndufa13 |  |  | 22241 | -0.281 | -0.1954 | Yes |
| 103 | 140520 | ATP8 |  |  | 22265 | -0.282 | -0.1931 | Yes |
| 104 | 436930 | uqcrc2a |  |  | 22306 | -0.284 | -0.1914 | Yes |
| 105 | 436968 | cox6b1 |  |  | 22427 | -0.291 | -0.1921 | Yes |
| 106 | 554135 | atp5b |  |  | 22579 | -0.300 | -0.1937 | Yes |
| 107 | 393884 | sdha |  |  | 22785 | -0.312 | -0.1969 | Yes |
| 108 | 336118 | cox7c |  |  | 22818 | -0.314 | -0.1946 | Yes |
| 109 | 449787 | atp5f1 |  |  | 22827 | -0.315 | -0.1915 | Yes |
| 110 | 553755 | atp5a1 |  |  | 22852 | -0.316 | -0.1889 | Yes |
| 111 | 108179059 | LOC108179059 |  |  | 23224 | -0.343 | -0.1970 | Yes |
| 112 | 767664 | cox7a1 |  |  | 23291 | -0.347 | -0.1954 | Yes |
| 113 | 393497 | ndufa11 |  |  | 23330 | -0.350 | -0.1929 | Yes |
| 114 | 445500 | sdhdb |  |  | 23455 | -0.358 | -0.1931 | Yes |
| 115 | 335025 | atp6v1g1 |  |  | 23477 | -0.359 | -0.1899 | Yes |
| 116 | 326975 | cox4i1 |  |  | 23518 | -0.362 | -0.1874 | Yes |
| 117 | 326962 | cox5ab |  |  | 23797 | -0.380 | -0.1921 | Yes |
| 118 | 402880 | cox4i1l |  |  | 23812 | -0.381 | -0.1885 | Yes |
| 119 | 550451 | ndufs3 |  |  | 23915 | -0.387 | -0.1877 | Yes |
| 120 | 393935 | atp6v1d |  |  | 24235 | -0.409 | -0.1934 | Yes |
| 121 | 394110\_1 | atp6v1aa |  |  | 24258 | -0.411 | -0.1898 | Yes |
| 122 | 554125 | ndufa2 |  |  | 24293 | -0.414 | -0.1865 | Yes |
| 123 | 322811\_1 | atp6v0d1 |  |  | 24364 | -0.415 | -0.1843 | Yes |
| 124 | 100002384 | cox5b1 |  |  | 24457 | -0.417 | -0.1828 | Yes |
| 125 | 337583\_1 | atp6v1ab |  |  | 24458 | -0.417 | -0.1784 | Yes |
| 126 | 445129\_2 | sdhc |  |  | 24579 | -0.420 | -0.1778 | Yes |
| 127 | 553672 | ndufs2 |  |  | 24748 | -0.434 | -0.1785 | Yes |
| 128 | 415253 | zgc:86599 |  |  | 24928 | -0.443 | -0.1794 | Yes |
| 129 | 100538220 | atp6v1c2 |  |  | 25059 | -0.451 | -0.1788 | Yes |
| 130 | 336957 | atp5c1 |  |  | 25134 | -0.456 | -0.1763 | Yes |
| 131 | 415158 | cycsb |  |  | 25213 | -0.461 | -0.1739 | Yes |
| 132 | 449655 | atp6v1c1b |  |  | 25334 | -0.469 | -0.1727 | Yes |
| 133 | 445129 | sdhc |  |  | 25446 | -0.479 | -0.1712 | Yes |
| 134 | 326977 | atp5l |  |  | 25518 | -0.486 | -0.1683 | Yes |
| 135 | 406301 | atp5g1 |  |  | 25665 | -0.500 | -0.1676 | Yes |
| 136 | 573689\_1 | ndufs4 |  |  | 25674 | -0.500 | -0.1626 | Yes |
| 137 | 359839\_1 | atp6v1ba |  |  | 25690 | -0.502 | -0.1578 | Yes |
| 138 | 103911376 | si:ch211-226l4.6 |  |  | 25780 | -0.510 | -0.1552 | Yes |
| 139 | 393776 | cox4i2 |  |  | 25810 | -0.512 | -0.1507 | Yes |
| 140 | 494577 | cox6c |  |  | 25868 | -0.517 | -0.1471 | Yes |
| 141 | 436799 | atp6v1f |  |  | 25896 | -0.520 | -0.1424 | Yes |
| 142 | 436768\_1 | uqcrq |  |  | 25910 | -0.521 | -0.1373 | Yes |
| 143 | 553691 | atp6v0a1b |  |  | 25976 | -0.527 | -0.1338 | Yes |
| 144 | 195824 | atp6ap1b |  |  | 25978 | -0.527 | -0.1283 | Yes |
| 145 | 493605 | ndufs1 |  |  | 25990 | -0.528 | -0.1231 | Yes |
| 146 | 557348 | cox8a |  |  | 26159 | -0.544 | -0.1226 | Yes |
| 147 | 192335 | atp6v1e1b |  |  | 26172 | -0.545 | -0.1173 | Yes |
| 148 | 286779 | atp6v1h |  |  | 26354 | -0.562 | -0.1170 | Yes |
| 149 | 335025\_1 | atp6v1g1 |  |  | 26589 | -0.587 | -0.1182 | Yes |
| 150 | 553767 | atpv0e2 |  |  | 26644 | -0.592 | -0.1137 | Yes |
| 151 | 797063\_1 | ndufb5 |  |  | 26691 | -0.598 | -0.1088 | Yes |
| 152 | 393720 | ndufv2 |  |  | 26805 | -0.610 | -0.1059 | Yes |
| 153 | 321951 | ndufa10 |  |  | 26930 | -0.623 | -0.1033 | Yes |
| 154 | 436768 | uqcrq |  |  | 26944 | -0.624 | -0.0971 | Yes |
| 155 | 541331 | ndufa9a |  |  | 26945 | -0.624 | -0.0905 | Yes |
| 156 | 402961 | ppa2 |  |  | 27207 | -0.645 | -0.0919 | Yes |
| 157 | 100333064 | uqcr10 |  |  | 27599 | -0.685 | -0.0970 | Yes |
| 158 | 556538 | ndufa12 |  |  | 27983 | -0.740 | -0.1013 | Yes |
| 159 | 554154 | uqcrb |  |  | 28106 | -0.757 | -0.0972 | Yes |
| 160 | 767750\_1 | atp5ib |  |  | 28490 | -0.818 | -0.1006 | Yes |
| 161 | 368907 | atp6v1c1a |  |  | 28719 | -0.863 | -0.0987 | Yes |
| 162 | 335751 | cox7a3 |  |  | 29214 | -0.977 | -0.1040 | Yes |
| 163 | 561117 | atp6v0a2b |  |  | 29956 | -1.226 | -0.1144 | Yes |
| 164 | 450017 | atp6v0e1 |  |  | 30042 | -1.263 | -0.1038 | Yes |
| 165 | 796997\_1 | ndufs7 |  |  | 30117 | -1.298 | -0.0924 | Yes |
| 166 | 573619\_1 | ndufab1a |  |  | 30417 | -1.458 | -0.0864 | Yes |
| 167 | 394181 | cox15 |  |  | 30485 | -1.497 | -0.0728 | Yes |
| 168 | 447914 | cox17 |  |  | 30691 | -1.664 | -0.0617 | Yes |
| 169 | 359840\_1 | atp6v1b2 |  |  | 31211 | -2.252 | -0.0543 | Yes |
| 170 | 393793\_1 | uqcrc1 |  |  | 31447 | -2.765 | -0.0325 | Yes |
| 171 | 447942\_1 | cox6a2 |  |  | 31777 | -4.401 | 0.0036 | Yes |
Table: GSEA details [plain text format]

  

Fig 2: OXIDATIVE\_PHOSPHORYLATION(DRE00190)      
 Blue-Pink O' Gram in the Space of the Analyzed GeneSet

  

Fig 3: OXIDATIVE\_PHOSPHORYLATION(DRE00190): Random ES distribution      
 Gene set null distribution of ES for **OXIDATIVE\_PHOSPHORYLATION(DRE00190)**
